# Supplementary material for: Are carbon emissions trading and green financial instruments synergistic? -Comprehensive quantitative research based on content analysis
Source: PLoS One. 2024 Mar 7;19(3):e0298601. doi: 10.1371/journal.pone.0298601 (PMC10919601; doi:10.1371/journal.pone.0298601)
Supplement: S1 Data — (ZIP) [file pone.0298601.s001.zip › Data/地级市Z_F工作报告.zip!/║╙─╧/σo╤⌠╩╨/└┤╫╘╣1⁄2╓┌║┼í╛┬φ┐╦╩2╛▌═°í┐_2010.docx]

各位代表：

　　现在，我代表市人民政府，向大会作工作报告，请予审议，并请政协各位委员提出意见。

　　 一、2009年政府工作回顾

　　 2009年，是新世纪以来我市经济社会发展最为困难的一年。面对国际金融危机的严重冲击和对我市的特殊影响，在省委、省政府和市委的正确领导下，全市上下深入学习实践科学发展观，认真贯彻落实中央、省应对国际金融危机的一系列政策措施，齐心协力，共克时艰，有效遏制了经济增速下滑的态势，全市经济形势企稳向好，各项工作在逆境中稳步推进，较好地完成了市六届人大一次会议确定的目标任务。

　　 ——经济形势企稳向好。初步核算，全市生产总值完成670亿元，增长10.5%。人均生产总值19097元，增长10.1%。地方财政一般预算收入25.34亿元、支出81.5亿元，分别增长0.1%和27.7%。规模以上工业增加值345.9亿元，增长12%。全社会固定资产投资434.6亿元，增长29.9%。社会消费品零售总额196.9亿元，增长18.5%。

　　 ——产业结构不断优化。一二三产业同比分别增长4.1%、11.4%和12.4%。三次产业比例由上年的13.8： 66.5： 19.7调整为14.0： 64.7： 21.3。

　　 ——人民生活继续改善。城镇新增就业7.6万人，城镇登记失业率2.23%，低于控制目标2.27个百分点。全市劳务输出68.6万人，实现劳务收入61亿元。城镇居民人均可支配收入13775.0元，农民人均纯收入4410.5元，分别增长8.2%、8.5%。

　　 ——社会大局和谐稳定。科技、教育、文化、卫生、体育等各项社会事业得到加强，信访、社会治安和安全生产形势总体平稳，社会大局稳定。

　　一年来，我们主要抓了以下重点工作：

　　 （一）战危机，经济保持平稳增长。把保持经济平稳较快发展作为经济工作的首要任务，及时采取一系列强有力措施，经济形势总体呈现企稳向好的态势。一是重点项目建设进展顺利。先后争取四批中央新增投资项目434个、资金4.1亿元，完成投资6.99亿元。列入省投资计划项目48个，完成投资61.5亿元。市定140个重点项目完成投资104.4亿元，占计划的115.7%，竣工项目105个。一批重大项目进展顺利。龙宇化工10万吨二甲醚、宏业化工2万吨清洁糠醛、力诺40万台太阳能热水器（一期）、金堤河张庄提排站改扩建、榆济天然气管道濮阳段等项目建成；濮范高速、大广高速南乐段、市城区至柳屯快速通道、大型商品粮基地等项目进展顺利；市工人文化宫、体育馆、蓝宝石温泉度假区等项目开工建设；投资14.4亿元的中原乙烯20万吨烯烃项目获中石化批复。我市荣获省重点项目建设投资目标先进单位。二是“三农”基础进一步巩固。认真落实支农惠农政策，发放涉农补贴资金4.3亿元。粮食总产达到249万吨，增长1.7%，再创历史新高。优质小麦、玉米、水稻播种面积占粮食种植总面积的80%。林下经济发展到15万亩，实现产值5亿元。畜牧业总产值63.4亿元，增长5.6%。市级以上农业产业化龙头企业发展到127家，实现销售收入92.6亿元，利税5.4亿元。实施土地综合整治项目22个，新增耕地6656亩，被授予全国基本农田保护先进单位。农田水利基本建设进一步加强，荣获全省“红旗渠精神杯”市级杯十连冠。解决了12.5万农民的饮水安全问题。新建农村户用沼气池3万座。新建、改建农村公路464公里。完成农村危房改造1780户。解决和巩固了4.9万贫困人口的温饱问题，被授予全省扶贫开发先进单位。新农村建设扎实推进，“争创产业强村、争当致富能手”活动成效明显，农民增收渠道不断拓宽；“四议两公开”工作法深入推广，村级民主管理机制进一步完善；新农村建设示范村典型带动能力不断增强。三是企业服务年活动成效明显。深入开展市、县领导干部集中服务企业等活动，帮助企业解决生产运行等方面的困难，重点企业生产经营好于预期。全年累计发生社会保险减负稳岗资金1.2亿元，帮助困难企业稳定就业岗位3.5万个。组织金融机构与企业签订合同186项，金额84.5亿元，到位资金43.3亿元。强化企业产销衔接，签订产销合同、协议金额2.9亿元。四是消费需求持续扩大。积极落实国家鼓励扩大消费的政策，兑现家电下乡、汽车摩托车下乡、汽车以旧换新等补贴资金6479万元，拉动消费3.2亿元。深入实施“万村千乡市场工程”，建成农家店546家。房地产开发完成投资35.97亿元，销售面积109.3万平方米。消费对经济增长的贡献率达到40%。五是外经外贸形势平稳。全市进出口总额3.97亿美元，其中出口3.23亿美元。外经三项指标均居全省前三位，荣获全省商务工作先进单位。六是财政金融平稳运行。面对国际金融危机严重冲击、国际原油价格大幅下跌、化工企业效益下滑和国家实施结构性减税政策带来的严峻挑战，财税部门通过优化服务，依法征管，财政收入自去年6月份开始好转，年底实现了止跌回升；通过加强预算管理，压缩行政开支，优化支出结构，保障了科技、教育、“三农”和民生等方面的刚性支出。金融机构各项存款余额525.6亿元，较年初增加82.2亿元。金融机构各项贷款余额198.4亿元，较年初增加43.5亿元，提前两个月完成新增贷款40亿元的省定目标任务，有力促进了地方经济发展。

　　 （二）调结构，可持续发展能力进一步增强。强力推进“一个载体、三个体系”建设，大力实施“以工兴市”战略，加快构筑濮阳科学发展的战略支撑平台。一是产业集聚区规划建设全面启动。全市8个省定产业集聚区总体规划、空间规划、控制性详细规划和规划环评通过省级评审。8个产业集聚区新开发面积5平方公里，完成基础设施和标准厂房投资19.8亿元；入驻规模以上企业20家，引进亿元以上投资项目11个。二是优势产业支撑能力不断增强。全市优势产业完成增加值305.3亿元，占规模以上工业增加值的88.3%。其中，石油化工、玻璃及电光源、林纸林板、食品加工等产业完成增加值256.6亿元，占优势产业增加值的84%。实施省 “双百计划”项目、省工业结构调整项目25个，完成投资33.9亿元。三是服务业成为新的经济增长点。市中心城区服务业园区被列为省服务业特色园区。我市被列为河南省文化改革发展试验区。编制了杂技产业发展规划。精心打造了《水秀》、《神龙部落》等精品杂技剧目。我市被中国杂技家协会命名为“中国杂技之乡”。免费开放了戚城文物景区、单拐冀鲁豫边区革命根据地旧址纪念馆。全年接待游客286万人次，旅游收入3.8亿元。全市服务业完成增加值142.8亿元，增幅较上年提高2.6个百分点。四是城镇化进程不断加快。全面推进第三轮城市总体规划实施。《濮阳科学发展示范区总体规划实施方案》报省待批。市县新一轮土地利用总体规划获省政府批复。加强城市基础设施和公共服务设施建设，市集中供热改扩建、地下水源给水工程、供水管网改扩建、燃气管网改扩建、公交综合保养场站、生活垃圾无害化处理场、污水处理厂脱氮改造、工业园区供水管网等基础设施（一期）重点项目进展顺利，10条背街小巷改造、“十站十厕”等项目全部完成。加强城市管理，大力开展综合整治，加强园林绿化工作，顺利通过“国家园林城市”复查验收和“国家卫生城市”省级年审。县城和小城镇建设取得新成绩。全市城镇化率达到35.43%，较上年提高1.65个百分点。五是科技创新能力不断增强。积极开展产学研合作，引进科技成果20项。实施重大科技专项6项。培育国家高新技术企业5家、国家级创新型试点企业1家、省级创新型企业2家、省级创新型试点企业7家。全市高新技术产业总产值133.2亿元，增长19%。六是节能减排和生态建设取得实效。严格实行节能减排问责制，加快淘汰落后生产能力。投资3.4亿元，实施节能改造项目27个。成功申报节能减排项目7个，争取奖励资金和项目扶持资金4919万元。全市建制镇垃圾集运设施建设全部完成。林业生态市建设扎实推进，全市林木覆盖率27.3%。单位生产总值能耗和化学需氧量、二氧化硫排放量均完成省定目标。

　　 （三）强动力，改革开放深入推进。坚定不移地深化改革开放，不断为濮阳发展注入动力和活力。一是大招商成效明显。出台《濮阳市招商引资优惠办法》等6个政策性文件，实行招商引资目标责任制，营造了较浓厚的招商氛围。组织参加第四届中博会等国家、省举办的大型招商引资活动，自主开展赴深圳、天津等专题招商活动，组织实施市级领导拜访央企、省企活动，成功举办“两节一会”经贸招商活动。全年共引进项目198个，总投资106亿元。利用市外资金86.7亿元，增长37.3%；实际利用外资7506万美元，增长541%，增幅居全省首位。二是各项改革有序推进。市属国有工业企业完成改革6户，盘活资产2.8亿元，安置职工4200人。市属国有非工业企业改革和县（区）国有企业改革取得阶段性成果。全市行政审批项目压减57项。我市被省确定为公立医院改革试点市。市城市信用社组建商业银行获中国银监会批复。市县两级政府机构改革、文化体制改革等各项改革有序推进。三是非公有制经济健康发展。全市规模以上非公有制企业895家，其中年营业收入超千万元的736家，超亿元的196家。全市非公有制经济完成增加值361.8亿元，占全市经济总量的54%。

　　 （四）惠民生，和谐濮阳扎实推进。高度重视改善和保障民生，切实解决涉及人民群众切身利益的热点难点问题。一是民生工程全面完成。筹措资金13.6亿元，落实省、市“十大实事”工程，年初对人民群众的承诺全部兑现。二是就业再就业和社会保障体系建设取得新成绩。失业人员再就业2.5万人，困难人员再就业1.4万人。购买公益就业岗位1.05万个。“零就业家庭”保持动态为零。8.7万返乡农民工就业问题基本解决。发放小额贷款4.92亿元，扶持创业10475人，带动就业25099人。企业退休、退职人员养老金人均月增115.48元。城镇职工和城镇居民基本医疗保险覆盖面分别达到99%和96%。积极推行城乡医疗救助制度，发放救助补助资金1485万元。全市实施廉租住房保障家庭6371户，惠及2.2万人。三是各项社会事业全面发展。落实城乡义务教育保障经费3.8亿元。完成了1541所中小学校舍安全排查鉴定和规划编制工作。普通高招本科上线率42.3%，居全省第一位。启动了职业教育攻坚计划。市城区社区卫生服务机构覆盖94%以上居民。投资1923万元，建设了一批中心卫生院和村卫生室。成功创建白内障无障碍市。甲型H1N1流感、手足口病等传染病防控取得成效。15个乡镇文化站、220个农家书屋建成投用。人口和计划生育工作取得新成绩，全市人口自然增长率控制在4.97‰。全民健身运动和竞技体育运动蓬勃开展。成功举办首届残运会。民族宗教、工商、质量技术监督、发展研究、外事侨务、机关事务管理、统计、史志、地震、气象、人防、保险、烟草、邮政、通信等工作都取得了新成绩。四是社会大局保持稳定。开展食品药品安全专项整治，产品质量和食品药品安全保障水平继续提升。安全生产形势稳定，连续六年被评为“全省安全生产工作优秀市”。健全应急管理机制，强化社会治安综合治理，做好信访工作，社会大局保持稳定。扎实开展国防教育，切实搞好民兵、预备役和人防队伍建设，支持驻濮解放军和武警、消防部队建设，被授予省级“双拥模范城”称号。

　　 （五）抓保障，民主法制和精神文明建设不断加强。加强政府法制工作，坚持依法行政，法治政府建设迈出新步伐。依法接受人大及其常委会的法律监督和工作监督，主动接受政协的民主监督，认真办理人大代表建议和政协委员提案。认真听取各民主党派、工商联、无党派人士的意见，支持工会、共青团、妇联等人民团体开展工作。切实加强精神文明建设，再次被命名为“全国创建文明城市工作先进城市”。认真开展深入学习实践科学发展观、“讲、树、促”活动，扎实推进“两转两提”，各级政府机关行政效能和服务水平进一步提升。加强行政监察和审计监督，反腐倡廉工作取得新成效。

　　 各位代表，过去的一年，我们在危机中坚定信心，在挑战中抢抓机遇，在困难中寻求突破，在逆势中团结奋进，经受住了严峻考验，在特殊年份实现了保增长、保民生、保稳定的目标。回顾应对危机的艰辛历程，我们深感成绩来之不易。这是省委、省政府和市委正确领导的结果，是人大、政协和社会各界有效监督、大力支持的结果，是全市人民和衷共济、艰苦奋斗的结果。在此，我代表市人民政府向全市工人、农民、知识分子、干部、人民解放军指战员、武警消防官兵、公安民警以及所有关心支持濮阳发展的各界人士致以崇高的敬意，表示衷心的感谢！

　　 在肯定成绩的同时，我们要清醒地看到，我市经济社会发展中还存在不少困难和问题。一是产业结构比较单一，过分依赖油气资源的结构性矛盾特别突出。二是经济增长质量不高，发展方式粗放，产业核心竞争力不强，财政收入未实现与GDP同步增长。三是重大项目谋划不足，储备不够，在建项目规模小，项目入驻条件差。四是有些干部忧患意识不强，工作作风不扎实；少数部门行政效率不高，服务意识不强。对此，我们要高度重视，并采取有效措施认真解决。

　　 二、2010年的经济形势

　　 2010年是实施“十一五”规划的最后一年，也是应对国际金融危机、为“十二五”发展打好基础的关键之年。综观国内外经济发展态势，总体上看，今年的经济发展环境好于去年，但面临的形势依然复杂。一方面，我们要看到，世界经济正在复苏，国内经济回升向好。国家继续实施积极的财政政策和适度宽松的货币政策，全面实施促进中部地区崛起规划，有利于我市争取更多的项目和资金，加强薄弱环节建设。沿海发达地区产业加快向内地转移，有利于我市开展招商选资、引技引智。我省实施十大产业调整振兴规划，有利于我市加快经济结构调整和产业优化升级。规划建设的八大产业集聚区，搭建了产业结构调整的载体。德商高速范县段、南林高速东延、山西中南部铁路通道濮阳段等重大交通项目纳入国家、省规划建设盘子，有利于构建我市连接环渤海经济圈和山东沿海地区的交通网络，建设好中原地区连接沿海发达地区的东大门。另一方面，我们也要看到国内外经济形势的复杂性。从宏观环境看，经济发展存在“三个不确定性”：一是外需能够恢复到何种程度不确定。虽然世界经济形势在缓慢好转，但实体经济回升仍面临较多困难。二是全球经济会不会再度出现局部性金融震荡不确定。虽然国际金融市场趋于稳定，但潜在的金融风险并未根本消除。三是通胀预期是否转化为现实通胀压力尚不确定。虽然我国经济呈现回升向好的势头，但经济发展中不稳定、不确定因素仍然很多。而且在后危机时期，我们既要满足保增长的要求，又要面对促转型的压力，工作中面临的“两难”问题明显增多。特别是，战危机保增长的实践，使我们更加深刻地认识到加快经济转型的重要性和紧迫性。我市石油、天然气及相关的化工产业占全市工业增加值的35%，占全部税收的近50%，占非农就业岗位的40%以上，随着油气产量的减少，以及受原油价格波动严重影响，我市可持续发展面临的压力越来越大。加快经济转型已经成为濮阳可持续发展的战略取向和必由之路。总之，只要我们准确把握经济发展走势，进一步增强忧患意识、责任意识、机遇意识和竞争意识，在应对挑战中树立信心，抢抓机遇，下大气力，下真功夫，就一定能够实现濮阳经济社会又好又快发展。

　　 三、2010年政府工作的总体要求和奋斗目标

　　 今年政府工作的总体要求是：全面贯彻落实党的十七大和十七届三中、四中全会精神，以邓小平理论和“三个代表”重要思想为指导，以科学发展观统领经济社会发展全局，按照省委、省政府“重在持续、重在提升、重在统筹、重在为民”的总体要求和“坚持三保、突出转型、强化态势”的战略部署，紧紧围绕加快资源型城市经济转型、实现经济社会可持续发展的战略任务，始终坚持以项目建设为抓手，以产业集聚区和综合交通体系建设为突破口，以保障民生为出发点和落脚点，强力推进工业化、城镇化、农业现代化协调发展，全面完成“十一五”规划确定的目标任务，努力促进濮阳经济社会平稳较快发展，为建设好中原地区连接沿海发达地区的东大门和加快资源型城市经济转型奠定更加坚实的基础。

　　 今年经济社会发展的主要预期目标是：生产总值增长10%；地方财政一般预算收入增长9%；全社会固定资产投资增长20%；社会消费品零售总额增长15%；外贸出口增长11%；实际利用外资增长10%；居民消费价格指数涨幅控制在3%左右；城镇居民人均可支配收入实际增长7%；农民人均纯收入实际增长6%；人口自然增长率控制在6.5‰以内；城镇新增就业5万人，城镇登记失业率控制在4.5%以内；单位生产总值能耗降低3.7%，化学需氧量、二氧化硫排放量分别下降2.34%和1.02%。

　　 四、2010年政府工作的主要任务

　　 （一）保持经济平稳较快发展，巩固经济持续企稳向好的态势。坚持把保持经济平稳较快发展作为经济工作的首要任务，落实完善扩大需求政策，强化调控措施，进一步巩固经济持续企稳向好的态势。一是保持投资较快增长。全年计划完成固定资产投资520亿元，其中工业投资完成300亿元，占总投资的57.7%；基础设施投资完成110.3亿元，占总投资的21.2%。初步安排重点项目150个，概算投资257亿元。其中超亿元项目36个，超5亿元项目14个，超10亿元项目8个。大力支持中原乙烯20万吨烯烃、中原大化20万吨乙二醇、河南永龙化工100万吨真空盐等项目建设。强力推进龙丰热电60万千瓦热电机组、龙丰纸业二期、中原大化气化岛等项目前期工作。完善和落实鼓励民间投资的政策，进一步拓宽民间投资渠道，在城建、教育、卫生、文化等10个领域集中推出一批项目，面向民间资本公开招标。设立专项资金，加强重点项目库建设，超前谋划和储备一批事关经济社会发展全局的大项目、好项目。落实联审联批、巡回观摩等措施，健全重点项目推进机制，加大项目推进力度。二是积极扩大消费需求。全面落实国家、省调整国民收入分配格局的政策措施，增强城乡居民特别是低收入群体的消费能力。落实最低工资制度。继续实施家电下乡、汽车摩托车下乡、汽车以旧换新补贴政策。建设农村太阳能热水器示范点，鼓励优先购买本地太阳能产品。继续实施“万村千乡市场工程”、“双百市场工程”和“农超对接工程”，依托供销、邮政机构，发展农村现代物流网络。加强和改善对房地产市场的调控，促进房地产业健康发展。积极培育信息、文化、旅游、健身、培训、家政、养老等消费热点。支持市移动公司、联通公司、电信公司加快建设3G网络等项目。进一步整顿和规范市场秩序，营造良好消费环境。三是深化服务企业活动。按照“深化服务、一企一议、突出实效”的工作思路，完善服务企业长效机制。市县两级分别抓好100家重点企业并实行动态管理。建立市县两级重点服务企业“信息直通车”制度。把服务企业的重点转向支持企业转型升级，促进企业加快技术创新和产品结构调整上来，不断延伸产业链条，提高市场竞争力。推进企业战略重组，培育大企业、大集团；加快建设中小企业公共服务平台、信息服务网络和创业基地，壮大、规范中小企业担保体系，大力支持中小企业发展。四是加强经济运行调控调节。加强煤电油气运供需协调，推进银企合作。加强市场监管，努力保持物价合理水平。优化财政支出结构，加大对“三农”、民生、节能环保等领域和社会事业的支持保障力度。完善社会综合治税机制，加强税收征管和非税收入管理，严厉打击涉税违法犯罪行为。落实各项税费优惠政策，减轻企业和居民负担。

　　 （二）夯实城市经济转型基础，促进经济社会可持续发展。坚持把加快经济发展方式转变作为深入贯彻落实科学发展观的战略举措，在发展中促转变，在转变中谋发展，夯实经济转型基础。一是坚持规划引领经济转型。深化对全局性、战略性的重大问题研究，编制好濮阳市“十二五”规划、濮阳市现代产业体系发展规划、濮阳市经济转型发展规划，确保年内完成。打破行政区划概念，立足三省交界区位优势，认真谋划区域经济发展战略，推进区域合作和经济协作，把濮阳打造成为中原地区向环渤海经济圈和山东沿海发达地区延伸、衔接、融合的平台。按照城市总体规划要求，加快完善各项控制性详细规划，使其覆盖率达到55%以上。完成城市综合交通规划、城市公交规划、城市绿地系统规划，启动并推进城市绿线规划、城市基础设施规划和城市风貌景观规划。二是加快产业集聚区建设。集中力量开展“产业集聚区建设加速年”活动，加快产业集聚区建设发展，打造产业结构调整的载体。完善基础设施。加快产业集聚区道路、供排水、供电、供气、污水处理和垃圾处理等公共基础设施建设。搞好产业集聚区土地储备，推广建设多层标准厂房，积极推进开发区科技工业城标准厂房建设，创造“土地等项目、厂房等项目”的招商优势。各产业集聚区完成基础设施投资3亿元以上，路网框架基本形成，标准化厂房面积达到2万平方米以上。厘清产业定位。坚定不移地按照“竞争力最强、成长性最好、关联度最高”的原则确定每个产业集聚区主导产业，大力引进同行业龙头企业和重大项目，大力引进一批关联度高、成长性强的中小型企业，形成各产业集聚区明晰的产业定位。每个产业集聚区完成产业投资10亿元以上，新培育3至5家销售收入超亿元的龙头企业。搭建服务平台。发展融资担保、现代物流、技能培训、科技研发、检测检验、信息咨询等生产性服务业，建设文化教育、医疗卫生、公寓餐饮等生活性服务业，增强产业集聚区配套服务能力。同时，落实省财政激励政策，执行直供电价。进一步理顺濮阳经济技术产业集聚区、濮阳市产业集聚区和濮东产业集聚区等管理体制。强化推进机制。继续实行产业集聚区建设“月例会、季观摩、年评比”工作制度，将产业集聚区建设纳入县级领导班子综合考评和政府目标管理考核体系，严格考评，扎实推进。市政府设立专项资金，采取“以奖代补”的形式，对发展好的产业集聚区进行奖励。三是推动产业结构优化升级。继续实施“以工兴市”战略，加快工业结构调整。按照“盘活做强存量，扩大提升增量”的原则，以重点支持龙头骨干企业做大做强为手段，着力打造“化工、玻璃及电光源、林纸林板、食品加工”四大产业基地，加快壮大优势战略支撑产业。以发展低碳经济为战略取向，以促进传统产业优化升级为抓手，加快培育机械设备制造、新材料、新能源、纺织等优势产业。进一步巩固和发展共生共赢的油地关系，围绕经济转型，油地共同谋划和实施一批重大项目。支持中原油田、中原乙烯等骨干企业做大做强，努力打造四个百亿企业。以推动集聚、加快提升、促进合作为途径，强力推进列入省“转型升级双千工程”的宏业化工15万吨过碳酸钠、贝英数控公司数控机床生产线等项目建设，调优产业结构。加快建设濮阳县濮耐集团柳屯项目三期、范县龙马实业60万芯千米通信电缆、南乐县宏业生化1.5万吨精制糠醛、台前县民通华瑞纸业10万吨优质轻量纸、新三强纺织20万锭纱等项目。同时，强力推进中原大化10万吨特种聚丙烯、龙宇化工航天炉扩能改造等项目前期工作。大力发展现代服务业，使其占GDP的比重达到22%以上。全面启动文化改革发展试验区规划建设，谋划和实施文化产业重大项目。着力打造特色鲜明、优势突出的文化旅游产品和精品旅游线路。重点扶持2个具备条件的村庄开展乡村特色旅游试点。举办濮阳国际精品杂技魔术节。对《水秀》进行二度创作，打造具有国际一流水准的精品力作。规划建设国内首家杂技主题公园。围绕中心城区服务业园区建设，规划包括油田商贸中心、联华购物广场等在内的核心商务圈。结合新的产业布局和交通枢纽布局，推进综合物流园区建设，加快推进市卷烟物流运输中心建设，加快粮食、医药、钢材等专业物流发展。加快濮阳市商业银行组建步伐，发展小额贷款公司。按照省统一部署，以县域为单位组建农村商业银行、农村合作银行。吸引外地金融保险机构在濮阳设立分支机构。积极推动蔚林化工上市，再培育5家上市后备企业。做好市长质量奖评选工作，引导企业树立品牌意识，提升产品质量和企业核心竞争力。争取开工建设市产品质量综合检测实验楼。四是加强综合交通体系建设。坚持交通先行，强力推进6条高速公路、2条铁路和5条快速通道建设。年内建成濮范高速、大广高速南乐段两条高速公路，开工建设德商高速范县段、南林高速东延两条高速公路，谋划濮阳至东明、濮阳至泰安两条高速公路，推进山西中南部铁路通道濮阳段建设，努力争取郑州至济南客运专线过境濮阳。年内建成市城区至柳屯快速通道，开工建设市城区至清丰县城、大广高速至南乐县城快速通道和范县新区至山东省界快速通道，谋划建设德商高速至台前县城快速通道，大力推进农村公路建设，着力打造综合交通体系。年内完成市长途汽车站改造，开工建设南乐县汽车站。五是提升自主创新能力。加强自主创新体系建设。围绕产业发展需要，组织实施一批重大科技专项。重点扶持5至6家企业组建市级工程技术研究中心，新培育2至3家省级工程技术研究中心。再培育3至5家河南省创新型试点企业、2至3家国家高新技术企业。组织实施产学研合作重点项目10项，新培育产学研合作创新示范企业2家。全面加强各类人才队伍建设，培养、引进高层次技术创新型骨干人才。六是抓好节能减排和生态环境建设。严格执行“两高”产业淘汰标准，加快淘汰落后产能。推广先进节能技术和节能设备。突出抓好电力、化工等重点高耗能行业的节能减排工作。积极开展建筑节能、交通节能和公共机构节能工作。完成省定集中供热分户计量改造任务。全面落实水环境生态补偿机制，开工建设环境监测监控中心，建成环境自动监控系统。继续推进林业生态市建设，完成林业生态工程造林8. 6万亩。巩固黏土砖瓦窑专项整治成果，坚决防止反弹。大力推进土地综合整治，落实城乡建设用地增减挂钩政策，坚持最严格的节约集约用地制度，推进闲置土地和低效利用土地开发和建设，提高土地综合利用效率。

　　 （三）统筹城乡发展，扎实推进城镇化进程。坚持把统筹城乡发展作为全面建设小康社会的根本要求，推动城镇化与新农村建设良性互动，逐步形成城乡经济社会发展一体化新格局。一是夯实“三农”发展基础。把解决好“三农”问题作为重中之重，全面贯彻中央一号文件精神，落实粮食直补、农资综合直补、良种补贴、农机具购置补贴和退耕还林补助等各项惠农政策，加大“三农”投入力度，促进农村发展、农业增效、农民增收。提高综合生产能力。认真组织实施粮食核心区建设规划，建成濮阳大型商品粮基地，提高农业综合生产能力，确保粮食总产稳定在240万吨左右。加强农田水利基本建设，抓好引黄干渠清淤疏浚，第三濮清南北延，渠村、彭楼、南小堤3个大型灌区续建配套与节水改造，马颊河清丰县段和台前县梁庙沟中小河流治理等重点工程。加快黄河标准化堤防工程建设。争取下半年开工建设南水北调支线配套工程。争取将金堤河干流二期工程纳入今年国家投资计划。坚持最严格的耕地保护制度，确保实现占补平衡。实施农业综合开发土地治理项目6.1万亩。加强高产田开发和中低产田改造。抓好国家级测土配方施肥试点县建设。加强基层农技推广体系建设，搞好濮阳县、清丰县农业科技创新体制改革试点工作。继续组织开展“万名科技人员包万村计划”。积极推进农机装备更新，加快农业机械化进程。建设特色产业基地。按照“一县一业、一乡一品”的原则，建设濮阳县和范县优质无公害水稻、濮阳县小杂果、清丰县小尖椒和食用菌、南乐县苹果和红杏、台前县无公害大蒜、高新区和华龙区无公害蔬菜及高档鲜切花等特色农产品基地。大力发展设施农业，以温棚蔬菜花卉为主导的各类高效经济作物发展到140万亩。大力发展现代畜牧业，建设生猪、奶牛、肉牛、家禽、肉羊等五大优质畜产品生产基地。完善基层动物防疫体系。积极推广林下养殖、林下种植模式，加快发展林下经济。提升产业化水平。大力支持农产品加工业发展，抓好雨润集团200万头生猪屠宰项目、河南大用集团肉鸡产业化、黄河滩区生态畜牧业示范项目建设，培育壮大127家市级以上农业产业化龙头企业，提高农业产业化经营水平。全市农业产业化龙头企业年销售收入超亿元的达到16家，超5亿元的达到2家；省级重点龙头企业达到20家，力争国家级重点龙头企业实现零的突破。推进农村改革。稳定和完善农村基本经营制度，加强土地经营权流转管理和服务。大力发展农民专业合作社，积极发展农业农村社会化服务组织，提高农业生产经营组织化程度。加快农村金融体制创新，推进农村信用社改革，积极培育村镇银行、贷款公司、农村资金互助社和小额贷款组织。基本完成集体林地明晰产权工作。加快县级供销社改革。完善新农村建设规划，增强新农村建设示范村带动能力，扎实推进新农村建设。二是提高城镇综合承载能力。发挥区域性中心城市的辐射带动作用。按照“产城互动、复合紧凑、融合发展”的要求，加快科学发展示范区（濮阳新区）规划编制，争取年内完成，并尽早争取省批准。从今年起，用三年时间基本打通城市断头路。年内打通长庆路、完成石化路西延。加快推进历山路、金堤路、振兴路、开州路、濮上路北延。通过打通长庆路，积极探索城建项目捆绑实施、封闭运行、整体推进、综合平衡的新模式。进一步完善土地储备制度和供地机制，盘活存量建设用地，通过土地储备和有序供地带动城市建设有序推进。加快城中村改造和旧城改造。实施“城市公共绿地建设工程”，年内建成德胜公园、濮水公园二期、颐和广场等公共绿地，提高人均公共绿地面积。继续抓好基础设施（一期）重点项目建设，确保年内按期完成；加快推进第二污水处理厂建设；推进濮阳供电公司濮阳东500千伏输变电工程及4个110千伏输变电工程前期工作；以启动濮阳平原水库规划建设为契机，做好城市水系规划，逐步形成濮阳城市水系景观，打造濮阳城市新亮点。以迎接国家卫生城市复检为契机，推进城市精细化管理，加快数字化城市建设，打造一至两条城市精细化管理示范路。加大城市综合整治力度，对城市违法用地和违法建设实行综合治理。加快推进市城区与濮阳县县城、清丰县县城一体化进程。以产业集聚区为载体，带动县域经济发展能力的提升，逐步将有条件的县城发展成为常住人口20万以上的中等城市。三是促进城乡协调发展。统筹城乡规划建设，年内全面完成县（区）村庄布局规划、乡镇总体规划和县域村镇体系规划编制，构建层次分明、结构合理的现代城镇体系。统筹城乡产业发展，推动城乡产业融合，逐步形成分工合理、产业对接、联动发展的格局。统筹城乡公共事业发展，促进城市基础设施和公共设施向农村延伸，推动城市教育、医疗等优质公共资源服务农村。推进城乡一体化改革，探索户籍管理、社会保障制度改革，有计划有步骤地解决农业转移人口在城镇的就业、子女就学、公共卫生和社会保障等方面问题，促进在城镇稳定就业和居住的农民有序转变为城镇居民。

　　（四）深化改革开放，增强经济社会发展的动力和活力。坚持把改革开放作为破解发展难题的根本途径，着力推进体制和机制创新，增强发展动力和活力。一是推进重点领域和关键环节的改革。积极引进战略投资者参与我市国有企业改革重组。积极稳妥推进林钢改制重组工作。全面启动市直机关所属企业脱钩改制。进一步完善国有资产监管体系。落实政策，放宽市场准入，大力支持、引导非公有制经济发展壮大。认真做好市县两级政府机构改革工作，确保按期完成改革任务。稳步推进事业单位分类改革和岗位管理制度改革。推进医药卫生体制改革，完善基本医疗保障制度，全面实现医疗救助与新农合、城镇职工和居民医保同步结算；搞好公立医院改革试点；逐步实施国家基本药物制度，初步建立基本药物供应保障体系；大力支持社会资本兴办医疗卫生机构。同时，按照省统一部署，扎实推进文化体制、财税体制、资源价格、政府投资等改革。二是扎实推进大招商活动。提高招商工作的针对性。紧盯国内外500强企业和行业龙头企业，寻求省与央企签署战略合作协议的新机遇，积极引进战略投资者，争取其项目优先布局我市。积极参加国家、省举办的重大招商活动。依托政府驻外机构，自主举办“长三角”、“珠三角”、“环渤海”经济圈等招商活动。立足资源优势，在我市举办“2010年中华张姓受封纪念大会”等专题招商活动。开展针对性强的小分队招商、定向招商和以商招商。建立签约项目跟踪问效机制，力促签约项目尽早落地，提高招商工作的实效性。三是主动拓展外部市场。抓住国际市场复苏和中原油田拓展外部市场的机遇，落实鼓励出口的各项政策，重点支持优势产品出口，扶持骨干出口企业发展，保持外经外贸工作的全省领先位次。年内完成商检办事处综合办公楼主体工程，海关联络点上半年投入运行。

　　 （五）抓好十项民生工程，努力促进社会和谐稳定。坚持把保障和改善民生作为发展经济的最终目的，着力解决事关群众切身利益的实际问题，促进社会和谐稳定。一是集中力量实施“十项民生工程”。（1）千方百计扩大就业。实现失业人员再就业1.2万人，其中帮助困难人员就业5000人，动态消除“零就业家庭”。新增转移就业农村劳动力2万人。继续实施扶贫培训“雨露计划”，培训贫困地区劳动力1.2万人。扩大小额担保贷款发放范围，全年发放小额担保贷款2亿元。（2）大力实施保障性安居工程。开工建设经济适用住房20万平方米，竣工10万平方米，为城市低收入住房困难家庭提供1200套经济适用住房；年内建成廉租住房5万平方米，为城市低收入家庭提供1000套廉租住房房源；新开工建设廉租住房1.7万平方米、340套。加大廉租住房租赁补贴发放力度，年底前完成保障户数5000户。改造农村危房2000户。（3）着力提高社会保障水平。按省定标准继续提高企业退休人员养老金。在清丰县开展新型农村社会养老保险试点，对60岁以上农村居民给予每人每月不低于60元的财政补贴。适当提高城乡低保标准，做到应保尽保。提高农村五保供养水平，分散供养年标准不低于1200元，集中供养年标准不低于2400元。加强法律援助，努力做到应援尽援。（4）着力促进农民增收脱贫。落实国家小麦收购政策，小麦最低收购价平均每斤提高0.03元。再解决4.65万农村贫困人口脱贫问题。完成100个贫困村的整村推进任务。（5）进一步改善农村生产生活条件。建设县乡公路150公里，改造危桥400延米。解决10万农村居民饮水安全问题。新增户用沼气池3000户，新建2处大中型沼气工程、10个乡村沼气服务站点。再建设一批农村连锁超市，覆盖全市90%以上的行政村，支持配送中心建设。新建300个行政村“村邮站”。（6）大力实施文化惠民工程。建成22个乡镇文化站、286个农家书屋。建成1个达到国家标准的文化信息共享工程县级支中心、1014个村级服务点。继续实施舞台艺术送农民工程和农村电影放映工程，为全市所有乡镇免费送一场演出，为全市行政村实施每村每月免费放映一场电影。支持市县艺术团体下乡演出。（7）优先发展教育。扎实推进中小学校舍安全工程。继续为全市农村中小学生免除学杂费和免费提供教科书，为城市中小学生免除学杂费，为农村家庭经济困难寄宿生提供生活费补助。完善助学制度，对中等职业学校在校一、二年级农村户籍、县镇非农户口以及城市家庭经济困难学生补助生活费，免除中等职业学校农村家庭经济困难学生和涉农专业学生学杂费。新建改扩建城镇中小学校10所，努力解决进城务工人员随迁子女入学和市城区学校大班额问题。（8）着力提高公共医疗卫生服务水平。开展免费婚前医学检查。全面实施新农合门诊统筹，将财政补助标准由80元提高到120元，最高补助封顶线由3万元提高到6万元。推进基本公共服务均等化，全市城乡居民人均基本公共卫生服务经费标准不低于15元。健全基层医疗卫生服务体系，改造1所县级医院，5所乡镇中心卫生院。实施农村卫生人才队伍建设“51111”工程，免费培训1600名注册乡村医生。继续实施扩大国家免疫规划，努力实现农村妇女在乡镇卫生院住院分娩（平产）全免费。（9）大力推进生态和城市基础设施建设。继续实施“节能产品惠民工程”，加大高效照明产品的推广普及，重点在城乡居民用户和县以下政府机构等单位推广。继续实施环境综合整治。对金堤河、马颊河等污染较重的流域，造纸、化工等污染较重行业进行综合治理。对地表水饮用水源地进行综合整治。落实农村环保“以奖代补”、“以奖促治”政策，创建16个省级生态文明村，3个省级环境优美小城镇。完成150个村（镇）绿化工程。改造背街小巷10条，建设59个乡的生活垃圾中转站，加快市生活垃圾无害化处理场项目建设。新建停车港湾60处、停车厅30处、灯箱式站牌30个，改造停车港湾16处。改造卫河市场燃气管道（人民路—胜利路）和15号热力交换站。（10）切实维护公共安全。全面启动“安全濮阳”创建活动，推动安全生产源头治理，继续强化危险化学品、烟花爆竹、建筑施工和石油天然气开采等重点领域整顿，开展道路交通和消防安全专项整治，坚决遏制重特大事故发生；继续开展食品药品安全专项整治，力争全市食品质量监督抽查合格率提高一个百分点。严厉打击食品生产领域制售假冒伪劣，及时查处食品质量安全违法违规案件。深化城乡平安建设，构建城市报警监控及农村技防体系。在此，需要说明的是，待省“十项民生工程”确定后，我市将及时对接，公布实施项目和具体内容，积极主动抓好落实。二是大力发展各项社会事业。大力推进素质教育。鼓励社会力量兴办教育。均衡发展义务教育。不断提升高中阶段教育、高等教育质量。大力推进职业教育攻坚计划，积极争创省级示范性中等职业学校和职业教育实训基地。实施好农村初中校舍改造、特殊教育学校建设等项目。年内完成市中医院病房楼主体工程，建成南乐县人民医院病房楼和范县人民医院病房楼，开工建设台前县人民医院病房楼。推进健康教育和卫生知识的普及。继续做好甲型H1N1流感等疫病防控工作。巩固白内障无障碍市创建成果，完善白内障复明工作长效机制。继续实施农村部分计划生育家庭奖励扶助和特别扶助制度，稳定低生育水平。繁荣发展文化事业，丰富群众文化生活。加强文艺精品创作，拍摄反映濮阳杂技发展历程的电视连续剧《百戏王》，排演大型现代戏《情系母亲河》。广泛开展全民健身活动。继续实施农民健身工程。加快推进市体育馆、博物馆和市工人文化宫建设。认真做好第六次人口普查工作。全面落实党的民族宗教政策，促进民族关系、宗教关系和谐。坚持统筹兼顾，做好发展研究、机关事务管理、外事侨务、统计、保险、地震、气象、史志、市场发展等工作。三是维护社会大局稳定。加强和改进信访工作，积极化解社会矛盾纠纷。完善应急管理机制，积极预防和妥善处置群体性事件和突发公共安全事件。加强社会治安综合治理，依法防范和严厉打击各类违法犯罪活动，确保社会大局稳定。四是加强精神文明和民主法制建设。深入开展社会主义核心价值观学习教育活动，大力弘扬焦裕禄精神、红旗渠精神和愚公移山精神。加强社会公德、职业道德、家庭美德、个人品德建设，深化大学生思想政治教育和未成年人思想道德教育。深入开展“扫黄打非”。 以创建全国文明城市为契机，广泛开展各个领域的精神文明创建活动，不断提高公民文明素质和城乡文明程度。推广“四议两公开”工作法，加强社区建设，完善村民自治。广泛听取各民主党派、工商联和无党派人士的意见，支持工会、共青团、妇联等人民团体积极参与社会管理和公共服务。继续加强国防教育和国防后备力量建设，支持驻濮解放军和武警、消防部队建设，不断提高民兵、预备役部队和人民防空建设质量；深入开展双拥共建活动，巩固发展军政军民团结。

　　 （六）加强政府自身建设，努力建设人民满意政府。坚持人民政府对人民负责、人民政府让人民满意。以开展效能建设活动为载体，扎实推进“两转两提”，加强政府自身建设，不断提高推动科学发展的能力和水平。一是牢记宗旨，增强忧患意识和责任意识。当前，我市处在经济转型的关键时期，面临保增长促转型的双重挑战。新形势新任务，要求我们必须时刻牢记党和人民的重托，进一步强化忧患意识和责任意识，紧紧抓住历史机遇，主动承担历史使命，以昂扬向上、积极进取、奋发有为的精神状态，化压力为动力，变挑战为机遇，将全心全意为人民服务的宗旨化为认真、出色履行岗位职责的具体行动，做到在其位、尽其责、谋其事，努力实现好、维护好、发展好人民群众的根本利益。二是转变作风，务求实干实效。坚持求真务实，善于把握规律，不断提高政府决策的科学性。要大力提倡调查研究，多深入基层，多深入群众，多深入一线，努力使掌握的社情民意更加准确、更加真实，努力使各项政策措施更加符合发展实际、更加符合群众利益。坚决反对形式主义、官僚主义，坚决避免急功近利、虚报浮夸。围绕确立的目标、部署的任务，扎扎实实抓落实，兢兢业业谋发展，确保政府工作定一件对一件，对一件干一件，干一件成一件，说到做到、说好做好，以正确的政绩造福于民，以良好的政风取信于民。三是提高效能，不断优化发展环境。深入实施“治庸计划”，不断强化服务意识，着力解决行政不想作为、不会作为、胡乱作为问题。加强对重大决策部署执行情况的监督检查，确保政令畅通。深化行政审批制度改革，进一步减少和规范行政审批事项。创新行政服务中心管理机制，做到一个机构对外、一个机构履职、一枚印章审批。加快电子政务建设，启动网上行政审批一期工程。建成市县两级行政效能电子监察系统，开展职能部门和科室长“双评议”活动，严格执行首问负责、限时办结、责任追究等制度，切实提高行政效能、工作效率和服务水平，为濮阳发展营造更加优良的环境。四是依法行政，做到廉洁从政。严格依照法定权限和程序履行政府职能。严格执行人大及其常委会的决议、决定，依法接受人大及其常委会的法律监督和工作监督，主动接受政协的民主监督，认真办理人大代表建议和政协委员提案。进一步加强政府法制工作，全面推行行政执法责任制，严格实行行政问责和行政执法责任追究。完善惩治和预防腐败体系，落实廉政建设责任制。坚决查处违法违纪案件。健全权力运行制约和监督机制，强化监察、审计等监督，依法推进政务公开。加强公务员队伍理想信念和廉洁自律教育，始终保持政府清正廉洁的良好形象。

　　 各位代表，发展为了人民，发展依靠人民，发展成果由人民共享。我们坚信，2010年必定是濮阳人民奋发图强、锐意进取、克服困难、实现新发展的一年！让我们在省委、省政府和市委的坚强领导下，以更加开放的胸怀、更加开阔的视野、更加扎实的作风、更加有力的措施，同心同德，开拓创新，扎实工作，奋力开创濮阳建设新局面，共同谱写濮阳发展新篇章！
